# Combined Before-and-After Workplace Intervention to Promote Healthy Lifestyles in Healthcare Workers (STI-VI Study): Short-Term Assessment

**DOI:** 10.3390/ijerph15092053

**Published:** 2018-09-19

**Authors:** Maria Luisa Scapellato, Vera Comiati, Alessandra Buja, Giulia Buttignol, Romina Valentini, Valentina Burati, Lucia La Serra, Isabella Maccà, Paola Mason, Pasquale Scopa, Anna Volpin, Andrea Trevisan, Paolo Spinella

**Affiliations:** 1 Department of Cardiac Thoracic Vascular Sciences and Public Health, University of Padova, Via Giustiniani 2, 35128 Padova, Italy; veracomiati@gmail.com (V.C.); alessandra.buja@unipd.it (A.B.); isabella.macca@unipd.it (I.M.); paola.mason.1@unipd.it (P.M.); pasqualescopa@yahoo.it (P.S.); anna.volpin@aopd.veneto.it (A.V.); andrea.trevisan@unipd.it (A.T.); 2 Preventive Medicine and Risk Assessment Unit, Padova University Hospital, 35128 Padova, Italy; giulia.buttignol@aopd.veneto.it (G.B.); lucia.laserra@aopd.veneto.it (L.L.S.); 3 Department of Medicine, University of Padova, 35128 Padova, Italy; romina.valentini@unipd.it (R.V.); valentina.burati.vb@gmail.com (V.B.); paolo.spinella@unipd.it (P.S.); 4 Dietetic and Clinical Nutrition Unit, Padova University Hospital, 35128 Padova, Italy

**Keywords:** health promotion, lifestyle changes, tailored counseling intervention, chronic diseases, work ability

## Abstract

Health care workers (HCWs) are prone to a heavy psycho-physical workload. Health promotion programs can help prevent the onset of chronic and work-related diseases. The aim of the STI-VI ‘before-and-after’ study, with assessments scheduled at 6 and 12 months, was to improve the lifestyle of HCWs with at least one cardiovascular risk factor. A tailored motivational counseling intervention, focusing on dietary habits and physical activity (PA) was administered to 167 HCWs (53 males; 114 females). BMI, waist circumference, blood pressure, and cholesterol, triglyceride, and blood glucose levels were measured before and after the intervention. The 6-month results (total sample and by gender) showed a marked effect on lifestyle: PA improved (+121.2 MET, *p* = 0.01), and diets became more similar to the Mediterranean model (+0.8, *p* < 0.001). BMI dropped (−0.2, *p* < 0.03), and waist circumference improved even more (−2.5 cm; *p* < 0.001). Other variables improved significantly: total and LDL cholesterol (−12.8 and −9.4 mg/dL, *p* < 0.001); systolic and diastolic blood pressure (−4.4 and −2.5 mmHg, *p* < 0.001); blood glucose (−1.5 mg/dL, *p* = 0.05); and triglycerides (significant only in women), (−8.7 mg/dL, *p* = 0.008); but HDL cholesterol levels dropped too. If consolidated at 12 months, these results indicate that our intervention can help HCWs maintain a healthy lifestyle and work ability.

## 1. Introduction

The gradual aging of the population [1], combined with a higher retirement age and a slower job turnover rate, inevitably leads to a significant aging of the workforce and an increase in workers’ burden of chronic disease. Older workers do not form a homogeneous group, however, and there can be considerable differences between people of the same age. The marked inter- and intra-individual variability can be associated with multiple factors (lifestyle, diet, physical activity, or genetic predisposition to disease, education, type of job, environment) that affect people’s health and the burden of chronic conditions in the adult population. Unhealthy behavior in daily life can be addressed with health promotion programs with a view to delaying and containing functional loss and the burden of chronic disease.

The WHO defines the workplace as an ideal setting and infrastructure for supporting health promotion for a large audience. Health promotion activities that introduce and support the maintenance of healthy lifestyles—providing appropriate information, counselling, and educational measures—need to be undertaken, and should preferably be an integral part of any Occupational Health and Safety (OHS) program [2]. Meanwhile, the Europe 2020 Strategy has specifically targeted employment, aiming to ensure that 75% of people aged 20–64 are working [3]. In fact, European citizens will have to work longer and longer, and it is therefore particularly important to implement programs to improve and promote the work ability of individuals and a healthy environment in the workplace [4,5,6,7]. Occupational physicians periodically meet workers individually and collectively, and are in an ideal position to serve as facilitators, taking advantage of multidisciplinary and multiprofessional approach oriented to disease prevention and health promotion [8].

Working in the health care professions seems to be particularly critical in this setting, due to the type of activities involved and to contingent needs relating to the current socio-economic environment, which expose health care workers (HCWs) to increasingly high physical and psychological demands [9]. In fact, Khamisa et al. [10] found that HCWs are at high risk of work-related stress and burnout, which is also associated with low satisfaction, lack of support, and poor physical and mental health outcomes.

The CERGAS Bocconi Report shows that the proportion of HCWs only partially fitness for work rises significantly with age [11]. This situation demands that we consider the worker as a whole, making an effort to prevent and assess the onset of diseases known to carry occupational as well as individual risk factors in their etiopathogenesis. Overweight and obesity, for example, are associated not only with some of the most common chronic conditions (metabolic syndrome, cardiovascular diseases, cancers) [4], but also with a higher risk of musculoskeletal disorders [12,13], which are the main cause of partial fitness for work among HCWs [14].

Another important element to consider is that healthcare staff are also seen as a key group for patients’ health promotion. It is therefore of utmost importance to implement programs to induce HCWs to exercise greater control over their own health (empowerment) and adopt lifestyles that serve as a positive example to patients too [15,16,17,18].

Evidence from the literature suggests that programs combining intervention (e.g., on diet and physical activity) with a multidisciplinary approach [4,19,20,21] obtain the best results in populations at risk, while focusing on a single aspect has a limited effect on long-term health outcomes [22].

Studies focusing on tailored behavioral interventions that use goal-setting techniques, individual counseling, and motivational interviewing, have proven more effective than general information and education sharing programs or supervised exercise [4,23].

To the best of our knowledge, such studies have never been proposed in Italian hospitals. On this basis, the Preventive Medicine Unit at Padova University Hospital (which deals with the health surveillance for workers exposed to occupational risks), in agreement with the hospital management, and in collaboration with the Dietetic and Clinical Nutrition Unit at the same hospital, launched a pilot health promotion project to improve employees’ lifestyles, taking advantage of the available human resources and in-house skills regarding nutritional science and counseling techniques.

Occupational Health services often have a health assistant (a specific Italian professional) on the team, but they are often employed more in activities to support occupational risk prevention than as counselors on health promotion programs. We believe that employing professionals specifically trained to manage such activities, together with other professionals (e.g., dietitians), could make a success of health promotion programs in the workplace.

### Aim

The purpose of this work was to conduct a ‘before-and-after’ study (STI-VI study) involving a short- and medium-term assessment (at six and twelve months) of a combined health promotion intervention that aimed to improve lifestyles (diet and physical activity) and metabolic and anthropometric measures of HCWs with at least one cardiovascular risk factor. This paper presents the results obtained after the first six months of a one-year intervention.

## 2. Materials and Methods

### 2.1. Study Design

The STI-VI study was a ‘before-and-after’ study involving assessments at the baseline (T0), and after 6 (T1) and 12 months (T2) of a combined intervention providing individual motivational counseling regarding physical activity and dietary recommendations.

The project was implemented at a large university hospital in northern Italy and participants have been informed and gave written consent to participate.

### 2.2. Sample

The study involved recruiting consecutive HCWs (on a voluntary basis) routinely examined by occupational physicians (OP) at the Health Surveillance Service of Padova University Hospital, who met the eligibility criteria outlined below. Enrollment, consent, and follow-up were completed between October 2016 and December 2017.

HCWs were invited to participate if they met at least one of the following criteria:

- overweight or obesity: a body mass index (BMI) > 25, or a waist circumference > 102 cm in men, or >88 cm in women;
- dyslipidemia: total cholesterol > 220 mg/dL, or high-density lipoprotein (HDL) cholesterol < 35 mg/dL, or low-density lipoprotein (LDL) cholesterol > 130 mg/dL, or triglycerides > 200 mg/dL, without pharmacological treatment;
- impaired fasting glucose (IFG) levels and/or impaired glucose tolerance (IGT), or diabetes mellitus, without pharmacological treatment.

The following exclusion criteria applied: refusal of written informed consent; diabetes mellitus under pharmacological treatment; hyperlipidemia under pharmacological treatment (statins and/or red rice yeast nutraceuticals); history of cardiovascular diseases; recent diagnosis of malignant tumors; pregnancy; other chronic illnesses, such as kidney failure, uncompensated endocrine disorders, etc. No age limits were applied.

**Figure 1:**
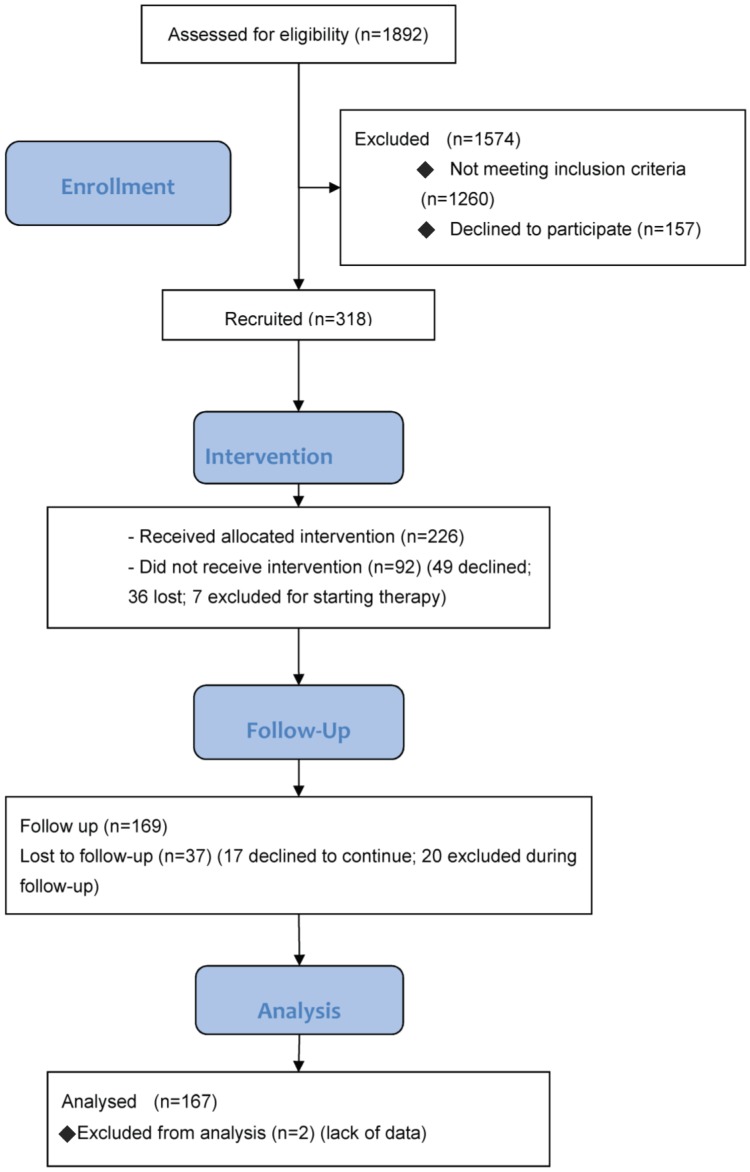
Flow chart and sample size.

Figure 1 outlines the STI-VI study procedure, participant flow, and sample size. About 1570 (83.2%) HCWs contacted were not recruited, largely because they did not meet the inclusion criteria, while 157 (3%) refused to participate, and another 157 (3%) met one or more exclusion criteria. The sample recruited for the study thus included 318 HCWs (16.8% of those contacted), and 226 (71.1%) of them were involved in the intervention. Of the 92 individuals recruited who were subsequently not involved in the intervention, 49 (53%) withdrew (generally due to shortage of time), 36 (39%) were lost during the study, and 7 (7.6%) started taking cholesterol-reducing therapy and were therefore excluded. Among the 226 involved in the intervention, 37 (16.3%) were lost to follow-up (at T1), and 20 discontinued the intervention, while 169 HCWs continued the intervention until the end. Two of these participants were excluded from the analysis because they did not attend the pathology lab for blood sampling, so the final sample analyzed consisted of 167 participants.

### 2.3. Measurements

At the baseline (T0), and after 6 (T1) and 12 months (T2), trained OPs measured and recorded the following variables during routine visits:

- current use of medication;
- physical activity: measured in terms of type, frequency (days/week), and duration (in minutes). To ascertain the energy expenditure for this physical activity, we converted these data into Metabolic Equivalent (MET) on the basis of the “Compendium of Physical Activities” [24]. Total calorie consumption was calculated using the International Physical Activity Questionnaire (IPAQ) [25] formula
  Σ (MET * frequency * duration)(1)
- blood pressure: systolic and diastolic blood pressure was measured three times on the left arm, with the subject seated and at rest for five minutes. The average of the second and third readings was recorded;
- waist circumference: measured to the nearest centimeter using a flexible steel tape, at the end of expiration, placing the tape on a level with the umbilicus [26];
- BMI: height and weight were measured with subjects barefoot and lightly dressed, and their BMI was calculated according to the formula: weight (kg) divided by height (m) squared;
- cholesterol (total, LDL, HDL), triglyceride and blood glucose levels: plasma total, LDL, and HDL cholesterol, triglycerides, and glucose were measured using standard enzymatic methods. For patients whose triglyceride levels were higher than 400 mg/dL, the LDL level was considered as missing.

They also collected from HCWs the following two instruments:

- a four-day Food and Physical Activity Diary [27]. Participants were given a food diary in which they were asked to report everything they ate and drank during two working days and two days off work within the week afterwards. They had to record quantities of food as faithfully as possible (in grams or standard portions) and any physical activity (type and duration). The data on participants’ diets were processed using the MètaDieta software approved by the ADI (Italian Association of Dietetics and Clinical Nutrition). This software runs calculations relating to food chemistry (quali-quantitative characterization), basal metabolism, BMI, energy requirements, and food portions. The software is based on official databases for the Italian population as at 2014 (INRAN—National Institute for Food and Nutrition Research—2008 revision; and LARN—Reference Nutrient and Energy Intake Levels) covering a total of 4500 foods and recipes, 114 bromatological components, and photographs of foods and recipes [28]. The analysis conducted on these data is not reported in the present paper;
- a food questionnaire from the PREDIMED trial. This Food Frequency Questionnaire (FFQ) contains 14 questions designed to assess the degree of adherence to the Mediterranean diet [29]. The questions investigate daily/weekly doses of nutrients such as fruit, vegetables, condiments (oil), meat, fats, etc., and generate a total score. The higher the score, the more the respondent’s eating habits come close to the Mediterranean model;
- at the time of the visit (T1 and T2), HCWs had to complete an anonymous satisfaction questionnaire containing four questions with yes/no answers, and the opportunity to add any comments/suggestions. The questions investigated whether the intervention had changed their lifestyles (Question 1), and sufficed to modify their eating habits and/or increase their physical activity levels (Question 2). Then there were specific questions about compliance with the timing of the meetings (Question 3), and whether the material received (food pyramid, brochure, etc.) had been adequate (Question 4). Participants were also asked whether the menus available at the workplace canteen favored their adherence to the dietary recommendations they had received.

### 2.4. Procedures

At the baseline—All HCWs invited to take part in the project (based on our specific inclusion and exclusion criteria) were informed about the study aims and procedures. If they were willing to participate, they provided their written informed consent and were given a summary of the study, with a list of scheduled meetings. OPs measured their anthropometric (weight, height, and waist circumference) and blood parameters, as described above, and gave the HCWs the Food and Physical Activity Diary and the FFQ to complete at home and return within a week for subsequent data processing.

Intervention—A month after their recruitment, HCWs attended an individual intervention during which they discussed the results of the Food and Physical Activity Diary, and the FFQ with the dietitian, who offered customized advice on their eating habits and level of physical activity.

On the same occasion, a health assistant from the Health Surveillance Service trained in motivational techniques encouraged participants to identify and set goals for their physical activity and proper nutrition. Using a motivational counselling approach, they were helped to identify strategies to achieve these goals, and given some helpful material, such as a food pyramid [30], and a brochure on physical exercise and reducing the use of salt in foods, published by the Italian Ministry of Health.

After three months, participants received further motivational support via phone counselling to remind them of their goals and how to change their lifestyle habits.

Follow-up—Six months after the intervention (T1), the HCWs were given an appointment with a resident in Occupational Medicine involved in the project, who collected a four-day Food and Physical Activity Diary and FFQ, newly completed at home, and measured anthropometric (weight, height, and waist) and blood parameters, as described above. The anonymous satisfaction questionnaire was distributed to the HCWs.

Participants were also asked about any difficulties they had experienced in changing their habits and any new pharmacological therapies they were taking.

The 12-month follow-up, using the same procedure as at 6 months, was still underway at the time of writing.

### 2.5. Statistical Analysis

The sample size was calculated on the basis of the waist circumference endpoint as a major predictor of cardio-metabolic risk factors [31]. A sample size of 148 achieves an 80% power to detect a mean 2 cm of paired differences, with an estimated 6.1 standard deviation of the differences and a significance level (alpha) of 0.05 using a two-sided paired *t*-test. Assuming a dropout rate of 10%, the minimum number of participants needed was 163 HCWs.

Descriptive analyses, in terms of mean, standard deviation, and percentages, were conducted for the variables investigated. Student’s *t*-test for paired samples was used to compare all assessment measures (anthropometry, nutrition habits, and physical activity) before and after the intervention (T0–T1). All comparisons were performed using two-tailed tests with a nominal significance level of 0.05. 95% confidence intervals for a proportion was computed.

The analyses were limited to participants with baseline data for all the different measurements. They were performed using STATA, version 14 (StataCorp. 2015. Stata Statistical Software: Release 14. College Station, TX: StataCorp LP, USA).

## 3. Results

### 3.1. Baseline Characteristics of the Sample

Table 1 shows the mean (and standard deviation) of the baseline variables, considering the whole sample stratified by gender. The average (median) age is quite high (50 years old) and does not differ between the two sexes.

Systolic blood pressure is high by AHA standards [32]; normal being under 130 mmHg (systolic); while diastolic blood pressure is slightly high; at over 80 mmHg. Considering the sample separately by gender; the men’s systolic and diastolic pressure levels are both ‘high blood pressure—stage 1’; while the women’s are lower pressure; with an almost normal diastolic pressure.

Waist circumference is 90.5 cm in the sample as a whole, but when considered by gender, men seem leaner than women (95.5 vs. 88.7). HCWs are generally overweight (BMI > 25), especially the women.

Total cholesterol levels are still within normal range (under 220 mg/dL), both overall and by gender. HDL are clearly high as a whole, and even higher in women. LDL are over the limit, with no differences between the two sexes. Triglyceride and blood glucose levels are within healthy range. Finally, total calorie consumption (indicated as ‘activity’ in Table 1) seems high overall, and much higher in men than in women.

### 3.2. Six-Month Follow-up

Table 2 and Table 3 show the differences in the variables ‘before-and-after’ the intervention in the whole sample, and by gender.

Regarding lifestyle, participants have increased their physical activity levels overall, though not to a significant degree in men. The participants’ dietary habits improved statistically overall, moving closer to the Mediterranean diet, with a one-unit increase in the medium score in the FFQ. The intervention prompted a weight loss, which is reflected in a substantial reduction in waist circumference (*p* < 0.001) and a smaller decrease in BMI; which is no longer significant when men are considered alone (*p* = 0.4). As for metabolic variables, the program led to an important reduction in total and LDL cholesterol (in the sample as a whole and by gender), but it led to a reduction in HDL cholesterol too. Triglyceride levels dropped overall, but only changed significantly in women. Blood glucose levels showed a small but significant reduction in the whole sample, and in women, with a smaller decrease in men. Both systolic and diastolic pressure measurements were lower, particularly in women, whose mean values after the intervention are within the normal range.

Among the HCWs who lost to the follow-up and stated the reasons for their withdrawal, most of them (63%) attributed their decision mainly to a shortage of time and too many work commitments; only one person reported having little faith in potential benefits of the program. When the drop-outs were compared with those who completed the six-month follow-up assessment, there were statistically significant differences in the baseline values for the following variables (expressed as mean ± SD): BMI (29.4 ± 5.4 vs. 27.1 ± 4.3, *p* < 0.001), waist circumference (95.4 ± 13.9 vs. 90.5 ± 12.0, *p* = 0.003), and age (47.3 ± 7.0 vs. 50.0 ± 7.3, *p* = 0.003).

**Figure 2:**
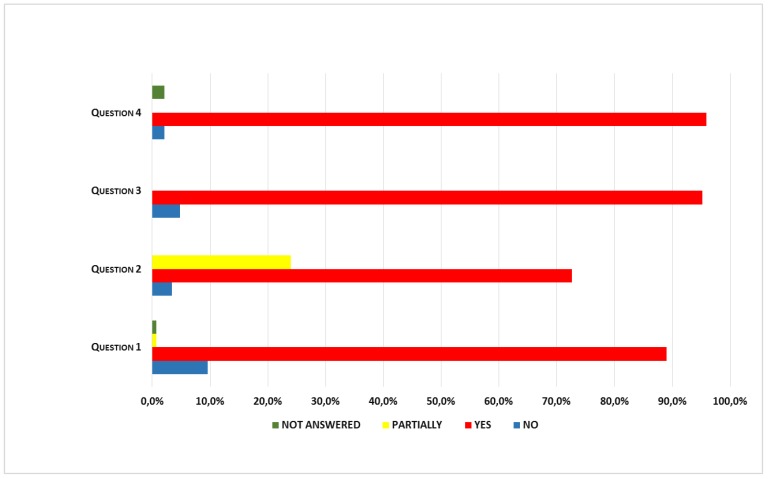
Results (% of answers) of satisfaction questionnaire submitted to participants.

Participants’ answers in the anonymous satisfaction questionnaire (see Materials and Methods for questions) are shown in Figure 2. Most participants answered all the questions and were satisfied with the intervention, feeling that it had led to a relevant change in their lifestyles. They also seem to have appreciated the supporting information material, and adherence to the schedule for the intervention. As for the menus offered by the hospital canteen, only 40% of the participants felt they favor adherence to the dietary recommendations received with the intervention.

## 4. Discussion

The health promotion program described here was characterized by a combined intervention on dietary habits and physical activity, which—according to the literature [19]—has proved the most effective approach.

The results of this study are promising and the intervention had a considerable effect on participants’ lifestyles. Their physical activity levels improved, and their eating habits have become more similar to the Mediterranean model. Their BMI became lower, albeit with a less evident improvement than for their waist circumference (this is consistent with other reports, however [33]). The metabolic variables showed an overall improvement, specifically total and LDL cholesterol (in both sexes), systolic and diastolic blood pressure and, with a significance improvement only in women, also triglyceride levels. A negative result emerged in the decrease in HDL cholesterol levels.

As a major predictor of cardio-metabolic risk factors, waist circumference probably showed the best improvement, as in other similar studies [34]. Christensen et al. [35] reported a major variation of −4.24 (*p* < 0.001), though the baseline characteristics of their sample point to a higher-risk population, as the initial medium waist circumference was 99.7 cm (98.7 cm in females), while in our sample it was 88.3 cm in women (and 90.5 for the HCWs as a whole). This difference in baseline values makes it difficult to compare the two results. Participants’ results in terms of waist circumference may be due to better dietary habits (more similar to the Mediterranean model after the intervention) or to an increase in physical exercise. Looking at the men’s results (Table 3), we find that a significant variation in waist circumference (−1.7 cm, *p* < 0.001) coincides with little change in physical activity levels, and a marked improvement in dietary habits. The result in waist circumference thus seems to be due almost exclusively to the newly-adopted more Mediterranean diet, with a higher intake of fruit, vegetables, and fiber, as also demonstrated by Mecca et al. [36]. We need to bear in mind that the men in our sample were already fairly active at the baseline, so the health assistant probably insisted more on encouraging them to set dietary goals during the intervention. On the other hand, the women in our sample started with lower levels of physical activity and succeeded in modifying both aspects of their lifestyle. Though the women showed an increase in their physical activity, they still exercise less than men and, when advised to exercise more, they typically answer that they are already physically very active at work. Most studies [37,38] show, however, that the biological effects on weight and cardiovascular risk of different types of physical exercise (sport as opposed to occupational activity) differ. It has been demonstrated that increasing the amount of activity at work is not associated with a reduction in BMI or cardiovascular risk [39]. A job involving higher levels of physical exertion can also have the negative effect of making people less inclined to exercise in their free time [40,41,42,43,44,45]. A greater physical demand at work can increase the sense of hunger too, prompting a higher calorie intake [38], and resulting in weight gain rather than loss. It would be important to provide detailed explanations about this aspect in future projects. As said before, the BMI reflects the reduction in waist circumference, even if the difference is not significant when men are considered alone. This may be due to their lower baseline BMI (26.6), which is almost within normal range, and/or to the smaller number of men in our sample. Some authors [35,36] found greater improvements in BMI than ours, but their studies were short-lived (10 weeks in one, three months in another), and—as the authors said themselves—it is physiological for any results achieved to dwindle with the passage of time.

As concerns metabolic factors, our intervention led to reductions in total and LDL cholesterol, triglycerides (significant only in women), and systolic and diastolic pressure. All these data are consistent with reports from Pattyn et al. [46], and Blackford et al. [47], and our results are far better than those reported by Groeneveld et al. [48], who found a medium drop of −2.2 in systolic, and −1.7 in diastolic blood pressure after 6 months (and this improvement tended to fade at 12 months).

Triglycerides deserve a separate mention. Ahmed et al. [49] reported that, even without any weight loss, a moderate increase in physical activity lowered triglyceride levels. Consistently with Blackford et al. [47], we found a concomitant improvement in physical activity and triglyceride levels in women (probably due to their greater number) and in the total sample, but no statistically significant change in these two factors in men. A meta-analysis [50] confirmed the influence of (aerobic) exercise on triglycerides, particularly on overweight or obese adults. In our sample, more women than men had a medium BMI and waist circumference that classified them as frankly overweight.

Lastly, other studies [48] reported variations in blood glucose levels after similar interventions, but this variation was generally not significant in general or higher-risk populations. In our sample, the initially apparent variation disappears when participants are divided by sex, probably due to the limited number of participants involved.

Strengths of our STI-VI project include its longitudinal design, and our combined and multidisciplinary intervention, exploiting the skills available at our university hospital. The involvement of personnel trained in counselling techniques at our Preventive Medicine Unit enabled a specific, tailored motivational counseling intervention to be implemented, which is what leads to the best and most lasting results according to the international literature [23]. Collaboration with the Dietetic and Clinical Nutrition Unit then assured us the nutritional and dietary expertise needed for such a study, so that participants would receive appropriate information on how to adjust their eating habits and lifestyles.

This study has some limitations. Although the overall results are positive, and largely reach statistical significance despite the small sample, the study suffered from high rates of dropout and participants lost to follow-up, which together amounted to almost 27% of the group initially recruited, and 22% during the follow-up (see Figure 1). This appears to be worse than for similar interventions: Groeneveld et al. [48] reported that 21.1% of their sample was lost to follow-up at six months; and Christensen et al. [35] reported quite a full adherence of the initial sample. On the other hand, when Jonsdottir et al. [15] performed a similar study in the public healthcare sector, they concluded that HCWs are less likely to participate in health promotion schemes than other populations. That said, our low adherence could also depend on the fact that ours is a pilot project, and the first to have been performed at our hospital.

To see whether the subjects lost to follow-up could have produced a sample selection bias, we checked for differences in the baseline characteristics between these subjects and those completing the follow-up. It emerged that the drop-outs were younger, and this might have contributed to their having a weaker perception of their cardiovascular risk, and not feeling the need to embark on a path like the one we proposed. On the other hand, the drop-outs were more overweight than those who continued the program, and might have been discouraged by the considerable amount of work that needed to be done. In fact, another study [4] showed that the best target population for workplace health programs includes people with a moderate cardiovascular risk, while such interventions are less effective in high-risk people (e.g., the obese). In our study too, the obese or more overweight subjects dropped out, thus giving rise to a selection bias. In the light of these observations, the findings concerning the efficacy of our program at six months should be interpreted with caution, as they mainly referred to older HCWs with a lower BMI. These observations regarding the HCWs who abandoned the study could also point to a challenge for future programs: appropriate strategies would be needed to improve adherence to such interventions focusing particularly on participants most in need, and therefore most likely to obtain physical and mental benefits [9]. Participants remaining in the study reported high levels of satisfaction: the anonymous questionnaire indicated that more than 90% of them were satisfied with the intervention and believe it had improved their lifestyles. When we investigated the drop-outs’ reasons for withdrawal, most of those who gave their reasons (orally or in writing) attributed their decision mainly to a shortage of time and too many work commitments. Only one person indicated a lack of conviction that the program could really help. We also recorded the reasons why some initially recruited HCWs were excluded from the program by the researcher (27 cases): 31.0% retired or moved to other hospitals; 4.7% started a pregnancy, 4.7% were diagnosed with serious health problems (e.g., cancer); 16.7% started cholesterol-lowering therapy; and 42.9% decided to embark on their own dietary path. Although this last reason led to participants being excluded from the study, it could be interpreted as the result of the study having a positive impact, since these participants became conscious of the need to change their lifestyle (and especially their diet).

Another limitation of the study lies in that participants may have overestimated their self-reported physical activity [51] and this may constitute a bias. All the other blood and anthropometric variables did not suffer from information bias, however, as they were measured objectively.

Furthermore, this study did not investigate the effect of physical activity on psychological conditions such as depression, which can lead to work-related disability and productivity loss [52]. In fact, physical activity can reduce the severity of depression as well as improving cardiac function [53]. The present study only focused on anthropometric and biochemical indicators, but the psychological aspect could certainly be investigated in a future study on HCWs.

## 5. Conclusions

The positive results identified six months after our combined intervention, although limited to the population in follow-up, encourage us to consider workers as a whole, and to deal not only with job-related risks but also with individual risk factors—very important in the etiology of many chronic and work-related diseases—to maintain their work ability as long as possible.

Our medium-term challenge will be to maintain these positive results at the 12-month follow-up too, by which time the benefits would be consolidated.

This pilot project, characterized by a tailored counseling intervention, exploiting the in-home skills available at our University Hospital, highlighted the importance of using a multidisciplinary approach in health promotion programs and the new role that OPs and more generally Occupational Health Services can play to improve HCWs’ health. This program could also encourage other Italian Hospitals to propose similar interventions aimed at improving the lifestyles of their employees.

## Figures and Tables

**Table 1:** Baseline characteristics for total sample, and by gender.

Variable	Total *N* = 167 Mean ± SD	Male *N* = 53 Mean ± SD	Female *N* = 114 Mean ± SD
Age (years)	50.0 ± 7.3	50.5 ± 8.3	49.7 ± 6.8
Pack year (*N*)	3.5 ± 8.4	4.2 ± 11.9	3.2 ± 6.1
Systolic BP (mmHg)	129.8 ± 13.4	137.3 ± 14.5	126.4 ± 11.3
Diastolic BP (mmHg)	83.0 ± 7.6	88.7 ± 7.2	80.4 ± 6.3
Waist circumference (cm)	90.5 ± 12.0	95.5 ± 10.5	88.3 ± 12.0
BMI (cm^2^/kg)	27.1 ± 4.3	26.6 ± 4.4	27.4 ± 4.2
Total cholesterol (mg/dL)	216.1 ± 32.0	213.8 ± 26.7	218.1 ± 34.0
HDL cholesterol (mg/dL)	58.9 ± 16.0	50.4 ± 14.0	63.2 ± 15.4
LDL cholesterol (mg/dL)	146.1 ± 28.5	147.2 ± 22.8	146.4 ± 30.5
Triglycerides (mg/dL)	113.8 ± 57.8	132.8 ± 71.2	104.7 ± 46.5
Glucose (mg/dL)	96.9 ± 10.6	99.8 ± 10.1	95.2 ± 10.4
Physical activity (MET)	497.3 ± 729.6 (2400; 7875) *	778.9 ± 1009.4 (4000; 1050) *	370.5 ± 519.0 (2250; 540) *

BP: blood pressure; BMI: body mass index; HDL: high density lipoprotein; LDL: low density lipoprotein. * median value; interquartile difference.

**Table 2:** Analysis on total sample before and after the intervention.

Variable	Before Mean ± SD	After Mean ± SD	Difference Mean ± SD	*p* Value
*Lifestyle factors*				
Physical activity (MET)	497.3 ± 729.6 (240.0; 787.5) *	619.6 ± 747.8 (402.5; 868.75) *	121.2 ± 600.4 (0; 337.5) *	0.01
Dietary	5.9 ± 1.6	6.8 ± 1.6	0.8 ± 1.7	<0.001
*Anthropometric factors*				
Waist circumference(cm)	90.5 ± 12.0	88.0 ± 11.5	−2.5 ± 4.3	<0.001
BMI (cm^2^/kg)	27.1 ± 4.3	26.9 ± 4.4	−0.2 ± 1.3	0.03
*Metabolic factors*				
Total cholesterol (mg/dL)	216.1 ± 31.9	203.9 ± 31.4	−12.8 ± 23.2	<0.001
HDL cholesterol (mg/dL)	58.9 ± 16.0	55.5 ± 14.5	−3.4 ± 8.0	<0.001
LDL cholesterol (mg/dL)	146.1 ± 28.5	137.3 ± 29.6	−9.4 ± 22.6	<0.001
Triglycerides (mg/dL)	113.8 ± 57.8	112.8 ± 89.0	−1.1 ± 85.5	0.87
Glucose (mg/dL)	96.9 ± 10.6	95.2 ± 9.6	−1.5 ± 9.9	0.05
Systolic BP (mmHg)	129.8 ± 13.4	125.4 ± 13.4	−4.4 ± 13.7	<0.001
Diastolic BP (mmHg)	83.0 ± 7.6	80.5 ± 8.7	−2.5 ± 8.8	<0.001

* median value; interquartile difference.

**Table 3:** Analysis before and after intervention, by gender.

Variable	Before Mean ± SD	After Mean ± SD	Difference Mean ± SD	*p* Value
*MALE* *Lifestyle factors*				
Physical activity (MET)	778.9 ± 1009.2 (400; 1050) *	860.7 ± 913.2 (600; 866.25) *	81.8 ± 673.8 (0; 390) *	0.4
Dietary (*N*)	5.6 ± 1.5	6.6 ± 1.9	0.9 ± 1.7	<0.001
*Anthropometric factors*				
Waist circumference (cm)	95.5 ± 10.5	93.7 ± 10.8	−1.7 ± 3.2	<0.001
BMI (cm^2^/kg)	26.6 ± 4.4	26.5 ± 4.6	−0.1 ± 0.1	0.4
*Metabolic factors*				
Total cholesterol (mg/dL)	213.8 ± 26.7	204.2 ± 28.1	−9.6 ± 19.8	<0.001
HDL cholesterol (mg/dL)	50.4 ± 14.0	47.7 ± 13.0	−2.7 ± 5.6	<0.001
LDL cholesterol (mg/dL)	147.2 ± 22.8	138.3 ± 29.8	−8.9 ± 23.9	0.009
Triglycerides (mg/dL)	132.8 ± 71.2	147.3 ± 138.9	14.5 ± 140.8	0.5
Glucose (mg/dL)	99.8 ± 10.1	98.3 ± 9.6	−1.6 ± 1.2	0.2
Systolic BP (mmHg)	137.3 ± 14.5	130.9 ± 15.0	−6.4 ± 15.3	0.004
Diastolic BP (mmHg)	88.7 ± 7.2	85.2 ± 9.2	−3.5 ± 9.0	0.007
*FEMALE* *Lifestyle factors*				
Physical activity (MET)	370.5 ± 519.0 (225, 540) *	509.6 ± 633.5 (360, 600) *	139.1 ± 566.1 (0, 330) *	0.01
Dietary (*N*)	6.1 ± 1.6	6.9 ± 1.5	0.8 ± 1.7	<0.001
*Anthropometric factors*				
Waist circumference (cm)	88.3 ± 12.0	85.4 ± 10.9	−2.9 ± 4.7	<0.001
BMI (cm^2^/kg)	27.4 ± 4.2	27.1 ± 4.4	−0.3 ± 1.5	0.04
*Metabolic factors*				
Total cholesterol (mg/dL)	218.1 ± 34.0	203.7 ± 33.1	−14.3 ± 24.6	<0.001
HDL cholesterol (mg/dL)	63.2 ± 15.4	59.3 ± 13.6	−3.8 ± 8.9	<0.001
LDL cholesterol (mg/dL)	146.4 ± 30.5	136.8 ± 29.6	−9.6 ± 22.1	<0.001
Triglycerides (mg/dL)	104.7 ± 46.5	95.9 ± 39.8	−8.7 ± 33.4	0.008
Glucose (mg/dL)	95.2 ± 10.4	93.7 ± 9.3	−1.5 ± 10.3	0.13
Systolic BP (mmHg)	126.4 ± 11.3	112.9 ± 11.8	−3.5 ± 12.9	0.005
Diastolic BP (mmHg)	80.4 ± 6.3	78.3 ± 7.5	−2.1 ± 8.7	0.02

* median value; interquartile difference.
